# Review of Who Are Dentists

**Published:** 1861-11

**Authors:** J. Allen


### REVIEW OF WHO ARE DENTISTS.

BY J. ALLEN, D. D. S.

[Continued from the August Number of the Dental Register.]

DESIROUS of correcting error in dental theory or practice, the writer will notice a few more points under the head of Who are Dentists, by Dr. Wm. A. Pease, as published in the Dental Register of the West. In referring to the different modes and materials employed for inserting artificial teeth, he presents what he conceives to be the merits and demerits of the various materials used as a base for that purpose, and his conclusions are that single gum teeth well mounted upon gold plate make the best practical artificial dentures that have yet been devised.

Although there are those who differ with him on this point, it is not proposed to discuss the question in this article, but simply give his own statements with reference to this (*best*) mode of setting artificial teeth; for his alternate views present all that need be said pro or con. In support of this style of work, he says: "It is not surprising that a metal so beautiful as gold, so incorruptible and plastic, and one withal of so great intrinsic value, should be considered almost instinctively by the profession and the community as one eminently suitable to be a base for artificial teeth. Alloyed and adulterated as it often has been, until it was sometimes difficult to determine whether it predominated over a mixture of silver and copper, it has nevertheless maintained its position in the professional and popular estimation, and it is believed that single gum teeth, well mounted upon gold plate, make the best practical artificial dentures that have yet been devised."

On the next page but one he spoils his golden picture by the following contra sentence: "A set of artificial teeth is a piece of mechanism—nothing more. It is made of light, frail and fracturable materials, and it is believed that the average duration of sets of artificial teeth will not exceed six years, while many of them are a constant source of trouble and expense from the first."

If this be true, it is certainly high time that a better method should be adopted. In reference to those who insert them, it will be recollected that Dr. Pease draws a line of distinction between those who operate upon the natural teeth and they who insert artificial ones. The one he calls *dentists*, and says "they are governed by the rules of a profession." The other, he states, "never rises above the customs of a *craft or trade*." Again, he says on page 292 Dental Register: "The country is now full of *dental mechanics*, and the facility with which they are manufactured in the laboratory, and the mistaken idea that dentistry affords lucrative employment is fast increasing the number; men are wanted in all the principal offices in the country to do the *office drudgery*, and make *artificial sets of teeth*." Now there are those who do not see this line of distinction as the Doctor does, and they say there are as many failures among those who operate upon the natural teeth, as there are with those who insert artificial ones, but the operations of the former are less conspicuous, and attract but little attention, while an artificial denture forms an important feature of the face, and is more noticeable. Incompetency on the part of those employed, in either branch, will defeat good practical results.

Again, referring to this (*best*) method, in comparison with continuous gum work, he loses sight of his sweeping denunciations against *all* who insert artificial teeth, and says on page 297, that "that kind of artificial teeth that can be made, and easily and cheaply repaired by all dentists, not only of a particular locality, but by all dentists of the country, is the safest, and therefore the *most desirable* and *valuable.*"

"Such are those mounted on *gold and silver ;* such are *not* those mounted on other materials, as they require a peculiar apparatus for their construction and repair, not used for other materials."

This universal method, which is adapted to the capacity of *all dentists*, he here recommends, although denounced in his preceding sentences, together with all those who construct them. Such incongruities are hard to reconcile, although he has endeavored to clothe them with plausible arguments. Still the coverings do not hide the apparent inconsistencies. These constitute one of the errors in his elaborate articles upon this subject.

Another error consists in attempting to depreciate an important branch of our profession, and of speaking disparagingly of *all* who practice in this branch ; for no good can result from such a course.

Another was in not confining himself to the question (who are dentists) and defining what constitutes a dentist, which he has not done, only in part.

Another occurs in commending his universality system, which he says "is the most *desirable* and *valuable*," because *all dentists* can make and repair this kind of work, which is made on gold and silver. Here, again, he seems to have forgotten what he had before stated, that these (*all*) dentists are not dentists, but mere mechanics, of which "the country is now full," and he seems also to have forgotten the miserable description he gave of their work in the April No. of the Dental Register. It is an old saying that "consistency is a jewel," but all do not see it.

Again his universality system is wrong, for it would tend to retard further advancement in dental science. According to this doctrine, no one must go forward, however emulous to excel in his profession, because all others would not advance simultaneously with him ; he must keep back and do the same style of work that every body else does, or be charged with a wrong, for departing from the old beaten track, although he might attain a better result by so doing.

But the Doctor says "a piece of mechanism, however valuable it may be, loses much of its value, if the means of repair are not *always* at hand." As well might it be said that the old mode of propelling boats was better than by steam power, because the means of repairing an engine were not always at hand, and the same may be said of any other improvement that has ever been made.

He is also cherishing an erroneous idea upon the subject of suction plates. He says that "the *weight of a set of teeth*, and the *pressure upon it* during mastication have to be *sustained by the tender mucous membrane* of the roof of the mouth, and is to that extent a constant and unnatural tax upon it, during life. Hence the lighter the material, the less is the strain or tax." There are also many others who entertain similar views, and they appear very plausible in the absence of facts. Now, we are all liable to err in theory, and in judgment, but in demonstration never. The *facts* do not sustain this theory ; a few pennyweights, more or less, in the weight of a set of teeth is not appreciable when in the mouth of the wearer, if the plate is properly fitted, for it will require at least twenty pounds beside the weight of the denture to dislodge it.

A plate well fitted to the mouth simply displaces the atmosphere from the membranous surface which it covers, and becomes an intervening substance between the atmosphere and the roof of the mouth; the atmospheric pressure then rests upon the artificial instead of the natural surface; but the pressure is no greater when resting upon the one than the other.

Again, when the atmospheric pressure is upon only one side of the plate in this manner, it overcomes the laws of gravitation, consequently there is *no weight* or *tax* upon the roof of the mouth. The same principle is easily demonstrated with other substances. Let two plates of glass be fitted so accurately as to expel all the air from between them, and it will be found that the atmospheric pressure upon the external surfaces will hold them firmly together; here there is no *delicate membrane* to sustain the weight or pressure.

The writer does not claim to be a lexicographer, but if he should attempt to define the legitimate sphere of a dentist, he would accord to him a more extended field of labor than merely operating upon the natural teeth; he would also embrace all other operations which require surgical skill, and therapeutic treatment, which often requires a high order of talent, not excelled, perhaps, in any other professsion, for complicated diseases connected with the teeth, jaws, bones of the face, and surrounding tissues, are often met with in dental practice, which require the best professional skill to overcome. He would also embrace all other operations that pertain to the artificial branch of dental science.

If a denture is to be inserted, the operator should possess artistic conception and harmonious execution, in order to adapt the shades, form, size, length and position of the teeth to the mouth, so as to produce perfect harmony with the other features of the patient, and also restore the original form and expression of the mouth and face. These essentials require well developed powers of mechanism, that the designs may be properly executed, however difficult to accomplish.

The foregoing form the chief qualifications of the gentlemanly dentist who is to preside over the whole field of dental practice.

